# Comparison between PtCO_2_ and PaCO_2_ and Derived Parameters in Heart Failure Patients during Exercise: A Preliminary Study

**DOI:** 10.3390/s21196666

**Published:** 2021-10-07

**Authors:** Mauro Contini, Alessandra Angelucci, Andrea Aliverti, Paola Gugliandolo, Beatrice Pezzuto, Giovanni Berna, Simona Romani, Calogero Claudio Tedesco, Piergiuseppe Agostoni

**Affiliations:** 1 Centro Cardiologico Monzino, IRCCS, 20138 Milan, Italy; mauro.contini@cardiologicomonzino.it (M.C.); paola.gugliandolo@ccfm.it (P.G.); beatrice.pezzuto@ccfm.it (B.P.); giovanni.berna@ccfm.it (G.B.); simona.romani@ccfm.it (S.R.); calogero.tedesco@ccfm.it (C.C.T.); piergiuseppe.agostoni@cardiologicomonzino.it (P.A.); 2 Dipartimento di Elettronica, Informazione e Bioingegneria, Politecnico di Milano, 20133 Milan, Italy; andrea.aliverti@polimi.it; 3 Cardiovascular Section, Department of Clinical Sciences and Community Health, University of Milano, 20122 Milan, Italy

**Keywords:** biomedical instrumentation, heart failure, exercise, transcutaneous PCO_2_, arterial PCO_2_, V_D_/V_T_

## Abstract

Evaluation of arterial carbon dioxide pressure (PaCO_2_) and dead space to tidal volume ratio (V_D_/V_T_) during exercise is important for the identification of exercise limitation causes in heart failure (HF). However, repeated sampling of arterial or arterialized ear lobe capillary blood may be clumsy. The aim of our study was to estimate PaCO_2_ by means of a non-invasive technique, transcutaneous PCO_2_ (PtCO_2_), and to verify the correlation between PtCO_2_ and PaCO_2_ and between their derived parameters, such as V_D_/V_T_, during exercise in HF patients. 29 cardiopulmonary exercise tests (CPET) performed on a bike with a ramp protocol aimed at achieving maximal effort in ≈10 min were analyzed. PaCO_2_ and PtCO_2_ values were collected at rest and every 2 min during active pedaling. The uncertainty of PCO_2_ and V_D_/V_T_ measurements were determined by analyzing the error between the two methods. The accuracy of PtCO_2_ measurements vs. PaCO_2_ decreases towards the end of exercise. Therefore, a correction to PtCO_2_ that keeps into account the time of the measurement was implemented with a multiple regression model. PtCO_2_ and V_D_/V_T_ changes at 6, 8 and 10 min vs. 2 min data were evaluated before and after PtCO_2_ correction. PtCO_2_ overestimates PaCO_2_ for high timestamps (median error 2.45, IQR −0.635–5.405, at 10 min vs. 2 min, *p*-value = 0.011), while the error is negligible after correction (median error 0.50, IQR = −2.21–3.19, *p*-value > 0.05). The correction allows removing differences also in PCO_2_ and V_D_/V_T_ changes. In HF patients PtCO_2_ is a reliable PaCO_2_ estimation at rest and at low exercise intensity. At high exercise intensity the overall response appears delayed but reproducible and the error can be overcome by mathematical modeling allowing an accurate estimation by PtCO_2_ of PaCO_2_ and V_D_/V_T_.

## 1. Introduction

Assessment of dead space/tidal volume ratio (V_D_/V_T_) and PaCO_2_ during exercise is of paramount importance for identification of exercise limitation at cardiopulmonary exercise testing (CPET) in the setting of several cardiovascular and pulmonary diseases, including heart failure (HF). V_D_/V_T_ value during exercise is calculated through simultaneous measurement of PaCO_2_ and mean expiratory PCO_2_ (P_E_CO_2_) [1]. V_D_/V_T_ is used to assess ventilation/perfusion mismatch [2,3] and it is elevated in case of concomitant pulmonary hypertension and/or respiratory disease. PaCO_2_ value during the isocapnic buffering period is a recognized index of reflex ventilation regulation [4,5]. Moreover, the end tidal CO_2_—arterial CO_2_ pressure gradient (ΔPetCO_2_ − PaCO_2_) during exercise is another parameter useful to assess ventilation perfusion mismatch in the lung [6]. 

Repeated arterial or capillary ear lobe blood sampling, both in static conditions and during exercise, to measure/estimate PaCO_2_ may be uneasy on a routine basis and outside of clinical settings. The technical reasons behind this are invasiveness, size of the catheter, instability of the calibration due to clotting, possible air contamination of the arterial blood sample and lack of reusability [7,8]. Therefore, non-invasive derived PaCO_2_ estimation seems desirable. End-tidal PCO_2_ (PetCO_2_) has been considered as a reliable estimate of arterial PCO_2_, in healthy subjects and in particular conditions such as monitoring during anesthesia [9,10]. However, increase in ventilation/perfusion mismatch makes evaluation of PaCO_2_ by PetCO_2_ highly unreliable in several diseases [11,12,13,14], as well as during sleep [15]. Moreover, a lack of accuracy in the estimation of PaCO_2_ and V_D_/V_T_ by PetCO_2_ has been reported during exercise making its use unreliable, at least in cardiorespiratory patients [16]. Regardless, albeit such an approximation is inaccurate, several commercial ergospirometers software report estimations of V_D_/V_T_ using PetCO_2_ as a PaCO_2_ surrogate [17].

Transcutaneous PCO_2_ (PtCO_2_) devices provide another option for the continuous noninvasive estimation of PaCO_2_, overcoming the limitations posed by end-tidal CO_2_ analysis [18]. PtCO_2_ is measured using Severinghaus–Stow-type electrodes, i.e., with an electrochemical sensor [19], with a heating system that brings the skin temperature up to about 42 °C. Commercial devices include probes with a single PtCO_2_ sensor, probes with a combination of partial pressure of oxygen (PO_2_) and PtCO_2_ and probes with a combination of pulse oximetry (SpO_2_) and PtCO_2_ measurements [20]. The methodology has been constantly improved over the years, making PtCO_2_ systems easier to use and more reliable in clinical practice. The main characteristics of commercial sensors are small dimensions (diameter 15 mm, height 8 mm), long-time for re-membranization (every 2 weeks), calibration required twice a day, short arterialization time (3 min) and high measurement reliability thanks to the protection of the membrane. This type of measurement has shown to closely approximate PaCO_2_ both at rest and during symptom limited exercise in normal subjects and in patients with lung disease [16]. Whether PtCO_2_ can be used as a reliable surrogate of PaCO_2_ during a maximal effort in patients with heart failure is actually unknown. The aim of our study was therefore to verify the correlation between PtCO_2_ and PaCO_2_ and between V_D_/V_T_ derived from PtCO_2_ and that derived from PaCO_2_ during a maximal exercise test in patients with stable heart failure. The novelty of this study consists in the application of such electrochemical sensors, which require stable operational conditions, in a highly dynamic situation where parameters are expected to change faster, and movement artefacts might be present. The final purpose of the study is to find an adequate protocol to use PtCO_2_ sensing on patients in dynamic conditions to estimate PaCO_2_ and derived parameters, such as V_D_/V_T_. Using such a protocol allows to study the response to exercise, i.e., to an increased metabolic demand, with a continuous sampling and a non-invasive method.

## 2. Materials and Methods

This study was designed as a sub study of a larger trial (Ethics Committee approval number CCM966) dedicated to the analysis of exercise performance in patients with severe HF. Study inclusion criteria were stable chronic heart failure with stabilized therapy in NYHA class II or III; left ventricular ejection fraction ≤ 40%, peak oxygen uptake (VO_2_) ≤ 12 mL/Kg/min or E/e’ > 13 at cardiac ultrasound and ability to perform a maximal exercise test on a bike. Exclusion criteria were age < 18 years, severe primitive valvular disease, significant pericardial disease, previous pulmonary embolism, peripheral arterial disease limiting exercise capacity, effort angina, or sign of ischemia at EKG, uncontrolled arrhythmias, pregnancy, severe pulmonary disease and the presence of any counterindication to exercise testing.

### 2.1. Procedures

A symptoms-limited maximal CPET was performed on a bike with a personalized ramp protocol (Quark PFT, Cosmed Cart, Rome, Italy) aimed to reach the maximal effort in around 10 min. The duration of the test was chosen based on the results by Agostoni et al. [21], who demonstrated that to assess exercise performance in HF patients by cardiopulmonary exercise test the exercise protocol needs to be 10 min long. To do so, the exercise protocol needs to be performed with a personalized progressive workload which means that the protocol must be adapted to the patient’s clinical conditions. Other types of protocols such as endurance or fixed workload or step increasing protocols do not allow to identify peak VO_2_. CPET was performed and analyzed following standard technique [22]. Briefly, patients were encouraged to continue the test until reaching a respiratory exchange ratio (RER) of at least 1.05. VO_2_, CO_2_ production (VCO_2_), PetCO_2_, end tidal oxygen pressure (PetO_2_), ventilation (VE), tidal volume (V_T_), respiratory rate (RR) and workload were recorded breath by breath and averaged every 10 s. Several minutes before the exercise test a small catheter was inserted in the radial artery. Arterial blood samples were collected at rest, i.e., beginning of the loaded pedaling, (minute 0) and every 2 min during exercise and immediately analyzed by a blood gas analyzer (GEM Premier 4000, Werfen, Barcelona, Spain) for PaCO_2_ determination. Heart rate (HR), hemoglobin O_2_ saturation (SpO_2_) and PtCO_2_ were monitored continuously. To measure PtCO_2_, a commercial electrochemical sensor combined with a heating system (V-sign™ Sensor 2, Sentec AG, Therwil, Switzerland) was used. The technical characteristics of the commercial measurement system used are reported in Table 1. 

The PCO_2_ measurement of the V-sign™ Sensor 2 is based on a Stow–Severinghaus type PCO_2_ sensor, i.e., a thin electrolyte layer is confined to the sensor surface with a hydrophobic, CO_2_ and O_2_ permeable membrane. The system was calibrated and applied over the patient’s earlobe through a clip with an adhesive layer after application of a fluid drop for optimal contact to the skin.

PtCO_2_ recording started after a stabilization time of around 10 min. A marker was applied at the time of arterial sample collection to synchronize PtCO_2_, PetCO_2_ and PaCO_2_ values. The V-sign™ Sensor 2 is known to have a response time <75 s, as reported in the user manual and in Table 1. Blood pressure was manually measured every two minutes by a sphygmomanometer. We analyzed data at rest, every two minutes of exercise and at the end of the ramp protocol. Since the loaded exercise started at 0 Watts no unloaded pedaling was done.

A total of 23 patients were enrolled in the present study, 6 of which have repeated the protocol twice, after treatment update. Overall, 29 acquisitions have been evaluated. The present study is a preliminary study, designed as a sub-study of a different research report. We were not able to define a priori the sample needed to define at each exercise step the reliability of PtCO_2_ measurements because no data exist to predict such a difference. For this reason, we used all measurements available from the original trial. The present report data may be used as reference for sample size determination on future studies on this topic.

### 2.2. Multiple Regression

**Figure 1:**
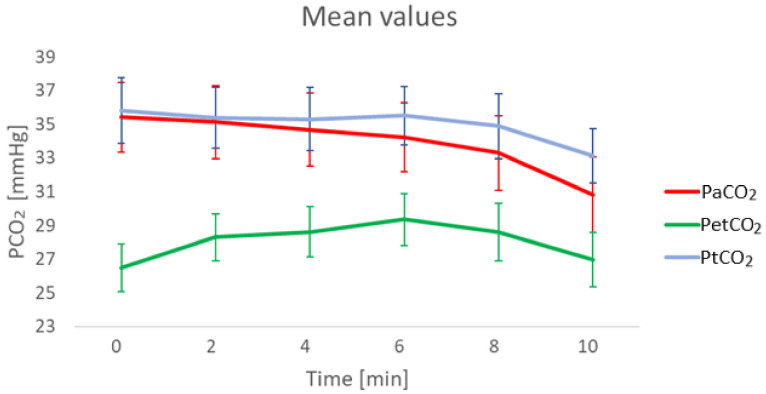
Mean values of PaCO_2_, PtCO_2_ and end-tidal CO_2_ partial pressure (PetCO_2_) at different timestamps. PetCO_2_ is shown for completeness of CO_2_ measurement parameters. 29 sets of data were available at minute 0, 2 and 4, 28 at min 6, 26 at min 8 and 22 at min 10.

As explained in detail in the following sections, it was observed (Figure 1) that the accuracy of PtCO_2_ measurements with respect to the reference PaCO_2_ values decreases at increasing timestamps, i.e., towards the end of the protocol. For this reason, the possibility of adding a correction to PtCO_2_ that keeps into account the time of the measurement during the protocol was exploited. 

This was implemented by means of a multiple regression model. Multiple regression is an extension of linear regression and is used to predict the value of a variable based on two or more inputs [23]; the general formula describing multiple regression is (1):

(1) $$\hat{Y}=b_{0}+\overset{m}{\underset{i=1}{\sum }}b_{i}X_{i}$$

where m is the number of input variables, $\hat{Y}$ is the predicted output, $b_{i}$ are the coefficients of the model ($b_{0}$ is the value when all input variables are zero) and $X_{i}$ are the input variables.

In this case, the value to be predicted is PaCO_2_ and the two variables that are used are PtCO_2_ and the timestamp (0 min, 2 min, 4 min, 6 min, 8 min, and 10 min). A corrected PtCO_2_ value is obtained afterwards, and this new value is compared to the previous results; the used formulation is given by (2):

(2) $$Corrected\;PtCO_{2}=f\left(PtCO_{2},t\right)=4.13-0.197\cdot \;\left(t-t_{0}\right)+0.873\cdot PtCO_{2}$$

In (2), t is a continuous variable expressed in min and $t_{0}$ is the beginning of the loaded pedaling in the ramp protocol. The function f is given by the multiple regression model, which was developed with a Python software based on the library *scikit-learn* [24].

### 2.3. Uncertainty of PtCO_2_ and V_D_/V_T_ Measurements

A first analysis consisted in estimating the uncertainty of PtCO_2_ and V_D_/V_T_ measurements obtained at different measuring times (0 min, 2 min, 4 min, 6 min, 8 min and 10 min) by analyzing the error between the two measurement methods: PaCO_2_ vs. PtCO_2_ and V_D_/V_T_ obtained from PaCO_2_ versus V_D_/V_T_ obtained from PtCO_2_.

Then, a multiple regression was applied to PtCO_2_ values at different timestamps to improve the measurements; the obtained values are referred to as corrected PtCO_2_. The formulas to compute the error of PtCO_2_ and corrected PtCO_2_ are, respectively, (3) and (4):

(3) $$Error\;before\;correction\;\left(t-t_{0}\right)=PtCO_{2}\left(t-t_{0}\right)-PaCO_{2}\left(t-t_{0}\right)\;$$

(4) $$Error\;after\;correction\;\left(t-t_{0}\right)=corrected\;PtCO_{2}\left(t-t_{0}\right)-PaCO_{2}\left(t-t_{0}\right)\;$$

where t is a continuous variable expressed in minutes, $t_{0}$ is the beginning of the loaded pedaling in the ramp protocol and their difference is the timestamp at which the measurement is performed. 

Bland–Altman plots [25,26], also known as Tukey mean-difference plots [27] in fields other than medicine and biosciences, were used to assess the agreement between the two measurement methods before and after the correction by multiple regression was applied. 

In the case of V_D_/V_T_, the estimation of the parameters is analyzed when each PCO_2_ value (arterial, non-corrected transcutaneous or corrected transcutaneous) is used, and the error is computed with the same method. In the present work, estimations of V_D_/V_T_ are performed with the Enghoff equation, i.e., using PaCO_2_, instead of the Bohr equation because the latter uses alveolar CO_2_ (PaCO_2_) [28]. $P_{E}{\mathrm{CO}}_{2}$ is the mean expiratory partial pressure of CO_2_ and was obtained from cardiopulmonary exercise testing (CPET). The formulas to obtain V_D_/V_T_ in the different cases are (5)–(7):

(5) $$\frac{V_{D}}{V_{T}}\left(t-t_{0}\right)\;from\;PaCO_{2}=1-\frac{P_{E}CO_{2}\left(t-t_{0}\right)}{PaCO_{2}\left(t-t_{0}\right)}\;$$

(6) $$\frac{V_{D}}{V_{T}}\left(t-t_{0}\right)\;from\;PtCO_{2}=1-\frac{P_{E}CO_{2}\left(t-t_{0}\right)}{PtCO_{2}\left(t-t_{0}\right)}\;$$

(7) $$\frac{V_{D}}{V_{T}}\left(t-t_{0}\right)\;from\;corrected\;PtCO_{2}=1-\frac{P_{E}CO_{2}\left(t-t_{0}\right)}{corrected\;PtCO_{2}\left(t-t_{0}\right)}\;$$

### 2.4. Analysis of the Deltas

Another characteristic that is important to evaluate is the ability of the transcutaneous measurement system to follow differential variations of the value during the protocol. For this reason, variations of the values at 6, 8, and 10 min with respect to 2 min have been evaluated before and after the correction of PtCO_2_ is applied. This was applied both in the case of PCO_2_ and V_D_/V_T_ extracted from transcutaneous measurements. The 2-min blood sample was chosen due to its lower variability.

The distributions of the deltas of PtCO_2_ and corrected PtCO_2_ are compared pairwise with the deltas of PaCO_2_; in the case of V_D_/V_T_, the deltas of the estimations obtained with PtCO_2_ and corrected PtCO_2_ are compared pairwise with the deltas of the estimations obtained from PaCO_2_. PCO_2_ deltas are expressed in mmHg, while V_D_/V_T_ deltas are adimensional.

### 2.5. Statistical Analysis

The distributions of the errors at different timestamps were analyzed with One-way repeated measurement ANOVA or an equivalent method for non-normal distributions. This method was chosen due to the repeated and correlated nature of the measurements. The comparison was performed considering minute 0 as the reference distribution in the case of PtCO_2_ and the derived V_D_/V_T_. 

The pairings of the deltas were first tested for normality with the Kolmogorov-Smirnov test [29]: for normal distributions, the paired t-test was used; for non-normal distributions, the Wilcoxon signed rank test was chosen instead.

## 3. Results

29 sets of data were available at minute 0, 2 and 4, 28 at minute 6, 26 at minute 8 and 22 at minute 10. Main clinical characteristics of the patients and main CPET results are reported in Table 2.

### 3.1. Uncertainty of PtCO_2_ Measurements

**Figure 2:**
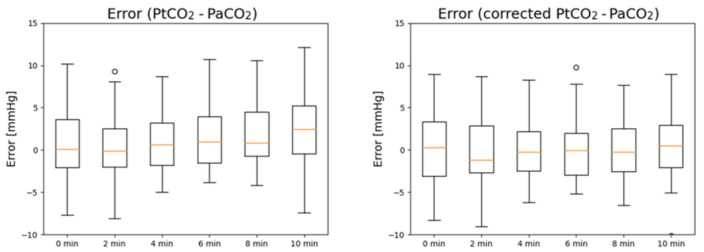
Boxplots of the measurement errors of PtCO_2_ (**left**) and corrected PtCO_2_ (**right**) at different timestamps with respect to PaCO_2_.

In Figure 2, the boxplots of the errors in PaCO_2_ estimation before (left) and after PtCO_2_ is corrected (right) are reported. PtCO_2_ without correction tends to overestimate PaCO_2_ for high timestamps, while the error is stably centered around 0 after correction is applied.

**Figure 3:**
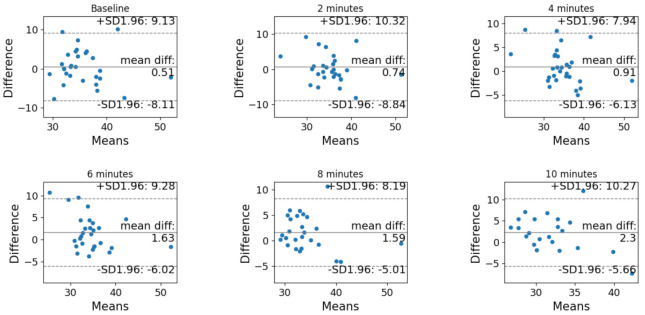
Bland–Altman analysis of the agreement between PtCO_2_ and PaCO_2_ before the correction at each timestamp (baseline, after 2 min, after 4 min, after 6 min, after 8 min and after 10 min).

**Figure 4:**
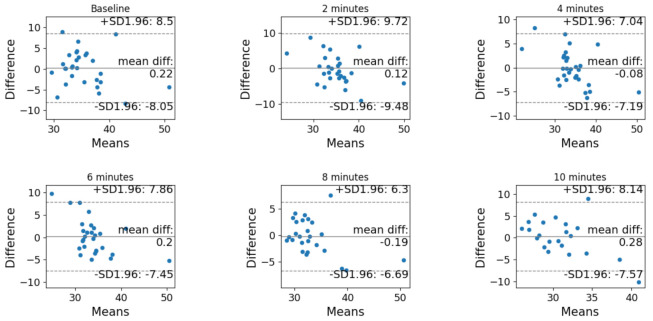
Bland–Altman analysis of the agreement between PtCO_2_ and PaCO_2_ after the correction at each timestamp (baseline, after 2 min, after 4 min, after 6 min, after 8 min and after 10 min).

The Bland–Altman analysis before and after correction is reported in Figure 3 and Figure 4, respectively. In both cases, the difference is computer as (non-corrected or corrected) PtCO_2_ minus PaCO_2_.

The results of the One-way repeated measurements ANOVA or equivalent test for non-normal distributions are different before and after the correction with multiple regressions is performed.

Before the correction, the distributions are not normal. According to the Bonferroni *t*-tests (multiple comparisons vs. a control group, in this case the baseline), the distribution of the error after 10 min is significantly different from the minute 0 distribution (*p* = 0.011), while the distributions at other timestamps (2, 4, 6 and 8 min) have a *p*-value > 0.05.

After the correction, the distributions are normal. The differences in the mean values among the groups are not great enough to exclude the possibility that the difference is due to random sampling variability; there is not a statistically significant difference (*p* = 0.978).

### 3.2. Uncertainty of V_D_/V_T_ Measurements Derived from PtCO_2_

**Figure 5:**
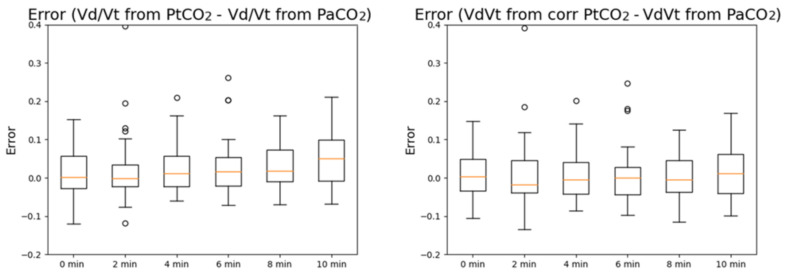
Boxplots of the measurement errors of V_D_/V_T_ computed with PtCO_2_ (**left**) and V_D_/V_T_ computed with the corrected PtCO_2_ (**right**) at different timestamps with respect to V_D_/V_T_ computed with PaCO_2_. The errors are expressed in mmHg.

In Figure 5, the boxplots of the errors in V_D_/V_T_ estimation from PtCO_2_ before (left) and after PtCO_2_ is corrected (right) are reported. Similar to PCO_2_, V_D_/V_T_ calculated with PtCO_2_ without correction tends to overestimate V_D_/V_T_ calculated with PaCO_2_ for high timestamps, while the error is stably centered around 0 after correction is applied.

Additionally in this case, the results of the One-way repeated measurements ANOVA or equivalent test for non-normal distributions are different before and after the correction of PtCO_2_ with multiple regressions is performed.

Before the correction, the distributions are not normal. According to the Bonferroni t-tests (multiple comparisons versus a control group, in this case the baseline), the distribution of the error after 10 min is significantly different from the minute 0 distribution (*p* < 0.001), while the distributions at other timestamps (2, 4, 6 and 8 min) have a *p*-value > 0.05.

After the correction, the distributions are still not normal. The differences in the mean values among the groups are not great enough to exclude the possibility that the difference is due to random sampling variability; there is not a statistically significant difference (*p* = 0.864).

### 3.3. Analysis of the Deltas

**Figure 6:**
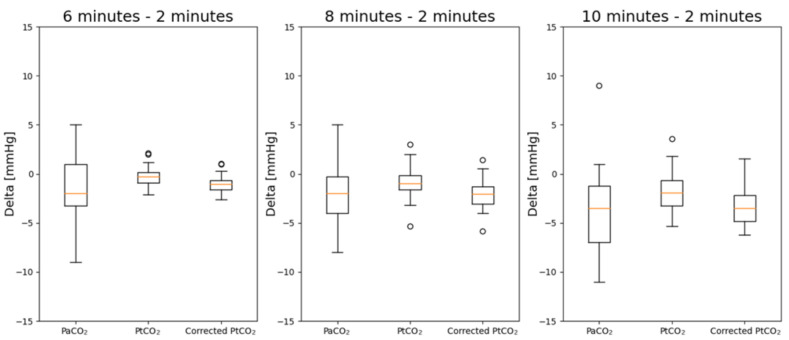
Boxplots of the deltas of PaCO_2_, PtCO_2_ and corrected PtCO_2_. The deltas are evaluated at 6, 8 and 10 min with respect to the values measured after 2 min.

In Figure 6, the deltas of PaCO_2_, PtCO_2_ and corrected PtCO_2_ at different timestamps with respect to the values measured after 2 min are reported. 

The results of the statistical analysis are reported in Table 3. Deltas with respect to 2 min have been considered. The deltas are evaluated at 6, 8 and 10 min with respect to the values measured after 2 min. 28 pairs of data were available at minute 6, 26 at minute 8 and 22 at minute 10. The values in italic are those where the distribution is not normal. The values in bold are those with a *p*-value < 0.05 which highlights a statistically significant difference. 

Without the correction by means of multiple regressions, there are statistically significant differences between the deltas at 8 and 10 min with respect to the measurements obtained after 2 min. The correction allows to remove such difference. Furthermore, the distributions after the correction are normal.

**Figure 7:**
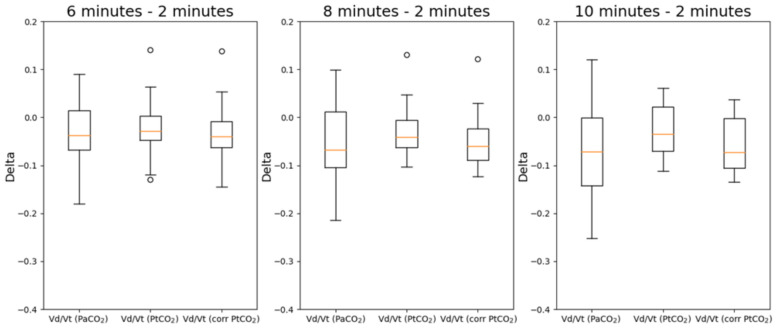
Boxplots of the deltas of V_D_/V_T_ estimated with PaCO_2_, PtCO_2_ and corrected PtCO_2_. The deltas are evaluated at 6, 8 and 10 min with respect to the values measured after 2 min.

The same analysis was repeated for V_D_/V_T_. In Figure 7, the deltas of V_D_/V_T_ estimated with PaCO_2_, PtCO_2_ and corrected PtCO_2_ at different timestamps with respect to the values measured after 2 min are reported while the overall results of the statistical analysis are reported in Table 4. The deltas are evaluated at 6, 8 and 10 min with respect to the values measured after 2 min. The values in bold are those with a *p*-value < 0.05 which highlights a statistically significant difference.

Deltas with respect to the value measured 2 min after the beginning of the protocol have been considered. In this case, all distributions are normal, so the only used test was the paired t-test. Without the correction by means of multiple regressions, there are statistically significant differences between the deltas at 8 and 10 min with respect to the measurements obtained after 2 min. The correction allows to remove such difference in all cases. 

## 4. Discussion

It is known from the literature that physiological parameters change with different levels of activity both inside and outside clinical settings [30,31] and that, at the same time, the performance of sensors and instrumentation in detecting these changes decreases with increasing levels of activity [32]. For this reason, it is relevant to study the performance of different types of instrumentation also during experimental protocols with highly dynamic activities and maximal exercise. The present study shows that in patients with severe but stable heart failure PtCO_2_ without any further correction is a reliable estimation of PaCO_2_ at rest and during a progressive workload exercise only at low exercise intensity. Indeed, when exercise effort increases and PaCO_2_ reduces the PCO_2_ value derived by transcutaneous sensors show a delayed response. This delayed response is partly due to the delay of the sensor system, which is known to be <75 s; however, it must be noted that even if the PtCO_2_ were partially shifted to take into account the delay, this correction would not be sufficient. Nevertheless, the overall response appears reproducible and therefore predictable, and the overall error can be overcome by mathematical modeling so that PtCO_2_ allow a precise estimation of PaCO_2_ and of PaCO_2_ derived data such as V_D_/V_T_. In brief our model allows to accurately estimate PaCO_2_ from PtCO_2_ during a maximal effort exercise and notably it allows to analyze exercise induced PaCO_2_ changes.

In terms of mathematical method, the presented article implemented a correction based on multiple regression. The implemented correction was performed by minimizing the error with a fitting on the existing samples, but if piecewise linearity is assumed the correction remains valid at any time sample between the beginning of the loaded pedaling of the ramp protocol and 10 min afterwards. The validity of the applied correction is therefore limited to this 10 min ramp protocol.

This correction method can be further improved by acquiring more data samples with the same technique and retraining the regression model accordingly. Another strategy could be in changing the timestamps for some subjects, for instance in sampling after 1, 3, 5, 7 and 9 min, to make the model more robust. Models that consider the correlated nature of such measurements could be also exploited, such as mixed-effects models. With an increasing number of subjects, however, it will be possible to use artificial intelligence techniques, such as strategies based on machine learning [33] and deep learning. In terms of time series forecasting, deep learning techniques are highly performing [34] and continuously improving; examples of possible methods include convolutional neural networks (CNNs), multilayer perceptions (MLPs) and long-short term memory (LSTM) networks. Accordingly, the present results, this study can be considered as a preliminary and feasibility analysis. 

The electrochemical sensor we tested for PtCO_2_ measurements needs heating of the earlobe skin up to a temperature of 42 °C. Consequently, it can only be applied for a relatively short time and not to prolonged measurements. No safety issue related to heating arose during the study protocol. However, in the literature, other strategies have been studied to measure transcutaneous blood gases and overcome the limitations of electrochemical sensors. As regards CO_2_ there have been recent projects attempting to measure PtCO_2_ with optical sensors, with a technology which is conceptually similar to pulse oximetry. These attempts include using an optical CO_2_ NDIR (non-dispersive infrared) sensor. Since CO_2_ gas reacts to 4.3 µm wavelength, this wavelength is selected using an optical filter before the sensor, so that only the presence of CO_2_ is detected [35]. With this technique, it might become possible to have more responsive sensors compared to electrochemical ones, thus better performances in continuous measurements with changes in parameters. This type of sensors could also be embedded in wearable devices, as it has been recently published by Tipparaju et al. [36], or garments [37] and integrated in telemedicine platforms [38], thus overcoming the limitations of measurements that are obtained in laboratory settings under the supervision of the clinician. Finally, optical sensors do not require any change of membrane, thus reducing the costs and making it possible to obtain also unsupervised measurements [7,20].

The present findings are a relevant step forward toward an extension in clinical practice of integrated exercise analysis which is needed for a better comprehension of exercise abnormalities [22]. Indeed, it allows to know PaCO_2_ dynamics during exercise without an arterial catheter otherwise needed for multiple sampling. At present, in the clinical field, direct or ear lobe PaCO_2_ are omitted or measured only immediately after the end of exercise limiting the observation of data only to peak exercise. Vice versa, PtCO_2_ continuous analysis allows to reliably estimate PaCO_2_ changes during exercise and precisely to obtain data at the anaerobic threshold, during the isocapnic buffering period as well as at the respiratory compensation point also known as the second ventilatory threshold. Indeed, PaCO_2_ data collected at these exercise steps provide relevant information about the chemoreflex regulation of ventilation and the causes of exercise induced hyperventilation which can be associated to V_D_/V_T_ as well as to reflex regulation [39]. As regards heart failure the information obtainable by PtCO_2_, and therefore PaCO_2_, V_D_/V_T_ and ΔPetCO_2_-PaCO_2_ allow to evaluate the possible presence of a concomitant lung disease, the reflex regulation of ventilation as well as the development during exercise of ventilation perfusion mismatch in the lung [6]. Moreover, the knowledge of PaCO_2_ values and derived data during exercise will allow a more personalized and efficacious heart failure therapy for examples as regards the choice of the of the most efficacious β-blocker in a specific patient [40].

Moreover, PtCO_2_ analysis seems to us a promising technique for future studies providing continuous information on CO_2_ changes and therefore to assess, much better than with repeated arterial blood sampling, CO_2_ dynamics, ventilation/perfusion mismatch, blood flow, through alveolar to earlobe transit time, and chemoreflex, through the CO_2_ value during the isocapnic buffering period. 

However, at present, a few limitations to the widespread use of the present technique must be acknowledge, such as the cost of the instrumentation, the time needed to heat the system before its use and the need of studies about its reliability and reproducibility in larger populations of patients with different heart failure etiologies and severity, as well as in patients with different diseases. Finally, our modeling has been built in ramp exercise protocol with expected exercise duration of approximately 10 min. We do not know whether it works in longer or shorter exercise or with different exercise protocols. A personalized exercise protocol with a progressively increasing workload built to achieve peak exercise in 10 min is considered the more physiologically correct and should be chosen in most cases [21,41]. Of note our model cannot be applied to other transcutaneous PCO_2_ transducers. To assess whether this method has clinical applicability, an external validation is required, such as a replication in a greater, different cohort. Furthermore, we studied a mainly male HF population. Accordingly, our results should be applied with caution in female patients at least before a dedicated study is done on female HF patients. Finally, as PtCO_2_ is largely affected by age, skin thickness, local temperature, usage of vasoactive drugs, poor tissue perfusion, and acidosis, the correlation between PtCO_2_ and PaCO_2_ might vary when these conditions exist: for this reason, our results should be applied only during exercise and in HF patients.

## 5. Conclusions

In heart failure patients PtCO_2_ is a reliable PaCO_2_ estimation at rest and at low exercise intensity. At high exercise intensity the overall response appears delayed but reproducible and the error can be overcome by mathematical modeling. In conclusion, during exercise PaCO_2_ and V_D_/V_T_ can be estimated from PtCO_2_ at rest and during a maximal workload exercise provided that a correction of a time delay is applied. A widespread use of this technique will likely enhance our knowledge in exercise physiology and allow a more personalized and efficacious patients assessment and, hopefully, treatment. 

## Figures and Tables

**Table 1:** Technical characteristics of the PtCO_2_ sensor as reported in the user manual.

Parameter	Value
Measurement range	0–200 mmHg (0–26.7 kPa)
Resolution	0.1 mmHg (0.01 kPa) below 100 mmHg (10 kPa) 1 mmHg (0.1 kPa) above 100 mmHg (10 kPa)
Drift	Typically < 0.5%/h
Response time (T90)	<75 s
Linearity	Typically < 1 mmHg (0.13 kPa)
Interference by anesthetic gases	Negligible
Stabilization/artifact detection	After sensor application or occurrence of a PtCO_2_ artifact, PtCO_2_ is displayed in grey until it (re)stabilizes

**Table 2:** Heart failure patient characteristics. Six subjects were tested twice. CPET data refer to 29 measurements. BMI = body mass index; CPET = cardiopulmonary exercise testing; VO_2_ = oxygen uptake; VE = ventilation; VCO_2_ = carbon dioxide production; RER = respiratory exchange ratio.

Characteristic	Mean ± SD
Age (years)	69 ± 8
Sex (M/F)	22/1
BMI	26.4 ± 4.6
Heart failure etiology (primitive/ischemic)	14/9
Ejection fraction (%)	27.5 ± 9.9
Atrial fibrillation (Yes/No)	11/12
Peak VO_2_ (ml/kg/min)	12.2 ± 3.7
Peak VO_2_ (%)	53 ± 15
VO_2_/Work slope (ml/min/watt)	7.9 ± 2.1
VE/VCO_2_ slope	43.7 ± 10.9
Peak RER	1.09 ± 0.09
Peak heart rate (bpm)	95 ± 25

**Table 3:** *p*-values after the pairwise comparisons of deltas of PtCO_2_ and corrected PtCO_2_ with respect to deltas of PaCO_2_.

	Delta after 6 min	Delta after 8 min	Delta after 10 min
PaCO_2_ vs. PtCO_2_	*0.099*	***0.022***	***0.023***
PaCO_2_ vs. corrected PtCO_2_	0.686	0.616	0.406

**Table 4:** *p*-values after the pairwise comparisons of deltas obtained with V_D_/V_T_ estimated with PtCO_2_ and corrected PtCO_2_ with respect to deltas of V_D_/V_T_ estimated with PaCO_2_.

	Delta after 6 min	Delta after 8 min	Delta after 10 min
V_D_/V_T_ estimated with PaCO_2_ vs. PtCO_2_	0.192	**0.037**	**0.008**
V_D_/V_T_ estimated with PaCO_2_ vs. corrected PtCO_2_	0.990	0.818	0.332
